# Solidarity and cost management: Swiss citizens' reasons for priorities regarding health insurance coverage

**DOI:** 10.1111/hex.12680

**Published:** 2018-04-14

**Authors:** Mélinée Schindler, Marion Danis, Susan D Goold, Samia A Hurst

**Affiliations:** ^1^ Institute for Ethics, History, and the Humanities Geneva University Medical School Geneva Switzerland; ^2^ Department of Bioethics National Institutes of Health Bethesda MD USA; ^3^ Department of General Internal Medicine University of Michigan Medical Center Ann Arbor MI USA

**Keywords:** health‐care policy, personal responsibility for health, priority‐setting, public involvement, solidarity, Switzerland

## Abstract

**Context:**

Approaches to priority‐setting for scarce resources have shifted to public deliberation as trade‐offs become more difficult. We report results of a qualitative analysis of public deliberation in Switzerland, a country with high health‐care costs, an individual health insurance mandate and a strong tradition of direct democracy with frequent votes related to health care.

**Methods:**

We adapted the Choosing Healthplans All Together (CHAT) tool, an exercise developed to transform complex health‐care allocation decisions into easily understandable choices, for use in Switzerland. We conducted focus groups in twelve Swiss cities, recruiting from a range of socio‐economic backgrounds in the three language regions.

**Findings:**

Participants developed strategic arguments based on the importance of basic coverage for all, and of cost‐benefit evaluation. They also expressed arguments relying on a principle of solidarity, in particular the importance of protection for vulnerable groups, and on the importance of medical care. They struggled with the place of personal responsibility in coverage decisions. In commenting on the exercise, participants found the degree of consensus despite differing opinions surprising and valuable.

**Conclusion:**

The Swiss population is particularly attentive to the costs of health care and means of reducing these costs. Swiss citizens are capable of making trade‐offs and setting priorities for complex health issues.

## 1 INTRODUCTION

Allocation decisions are pressing in all health systems, yet determining priorities for limited health resources is a difficult task often fraught with disagreement. This has led to endorsement of procedural approaches to priority‐setting^1^ and to calls for public participation in priority‐setting.^2^ Procedural approaches have, however, also been criticized as failing to achieve true public involvement^3^ and failing to avoid the disagreements between reasonable people that arise when implementing substantive principles for priority‐setting.^4^ Moreover, public involvement in coverage decisions tends to focus either on general principles or on specific interventions, rather than addressing the trade‐offs required by priority‐setting.^3^ Empirical research exploring the views of the public and their ability to deliberate, reach consensus, and provide utilizable direction on priority‐setting in health care, can offer valuable information about the feasibility of such approaches. Previous studies of public attitudes in the United States have shown that deliberation on priorities in health care leads citizens to increase priority to the uninsured,^5^ prioritize socio‐economic determinants of health^6^ and have led to increased participant willingness to abide by group decisions even when they had made different choices themselves.^7^

We conducted a study of public deliberation for health‐care priority‐setting in Switzerland, a country with high health‐care costs, universal coverage through an individual health insurance mandate and a strong tradition of direct democracy with frequent votes related to health care.^8^ The Swiss health‐care system is not a National Health Service, but one in which individual choice is central. The Swiss tradition of democratic deliberation results in a population familiar with frequent participation in policy decisions, who votes on issues related to health care on a regular basis. Given the rising cost of health insurance, how would this population make trade‐offs between competing needs? What sorts of limits on access to services would or would not be acceptable? How would Swiss citizens balance public good and social responsibility with individual needs and preferences? In this study, we focus on the values and justifications citizens used during deliberations about priorities in health‐care coverage.

## 2 METHODS

### 2.1 Adapting the deliberation tool

We adapted the Choosing Healthplans All Together (CHAT)^1^ exercise that has been previously developed for similar exercises in the United States, New Zealand and India.^9^, ^10^, ^11^ This is a simulation exercise based on a serious game enabling participants to put together a health insurance package and thus express priorities and trade‐offs. Initial development of this exercise has been described elsewhere.^12^ Adapting the CHAT tool for use in Switzerland involved four steps: (i) identification of the most relevant questions, (ii) modifying the exercise materials for Switzerland, (iii) developing scenarios fitting the Swiss health‐care system and (iv) translation into local languages. To identify relevant questions, we held preparatory discussions with Swiss “key informants” expert people on health financial implications: physicians, politicians and patient representatives involved in issues regarding the health‐care system. Based on these discussions, we included options designed to assess attitudes regarding aspects of health‐care financing such as the level of co‐pay or premium subsidies. To design trade‐offs based on realistic scenarios, we worked with Milliman, an international actuarial company experienced with adaptations of the CHAT project for different US states, to create insurance benefit options that would be compatible with the Swiss health‐care system and relevant there. We then created scenarios—or health events—to help participants think about and appreciate the practical consequences of their benefit choices. Finally, we translated the material into German, French and Italian. Translations were back‐translated and checked by individuals familiar with these languages.

### 2.2 The Swiss health‐care system

Switzerland's health system reflects the federal structure of the country. It is based on an individual mandate for insurance covering a federally defined basic package. Basic insurance is provided by dozens of private health insurance funds, with the 10 largest insurers covering over 80% of policyholders. Coverage for services included in insurance packages is 90% of the cost above the deductible in Swiss francs (300‐2500 CHF), with co‐pays capped at 700 CHF per year for adults, and 350 CHF for children. Provision of care is organized by the 26 cantons. The confederation thus guarantees a health system where everyone must be affiliated and covered to the level of basic health insurance. Premiums vary with the canton of residence, but cannot be risk‐adjusted in other ways.

### 2.3 Participants

Participants were recruited throughout Switzerland through a market research agency (Yxplora, Zürich). To illustrate the diversity of organizational and cultural aspects within our health‐care system, volunteering participants were selected based on five stratification criteria: rural or urban, gender, age, socio‐economic level and language. We were not able to selectively recruit healthy and sick persons given the private nature of that information. To recruit as broadly as possible geographically, we conducted focus groups in four French‐speaking cities (Geneva, Lausanne, Bienne and Sion), six German‐speaking ones (Bern, Basel, Zurich, St‐Gallen, Chur and Luzern) and two Italian‐speaking ones (Lugano and Bellinzona), recruiting each time to include participants from both rural and urban areas.

### 2.4 Deliberative exercise

**Figure 1:**
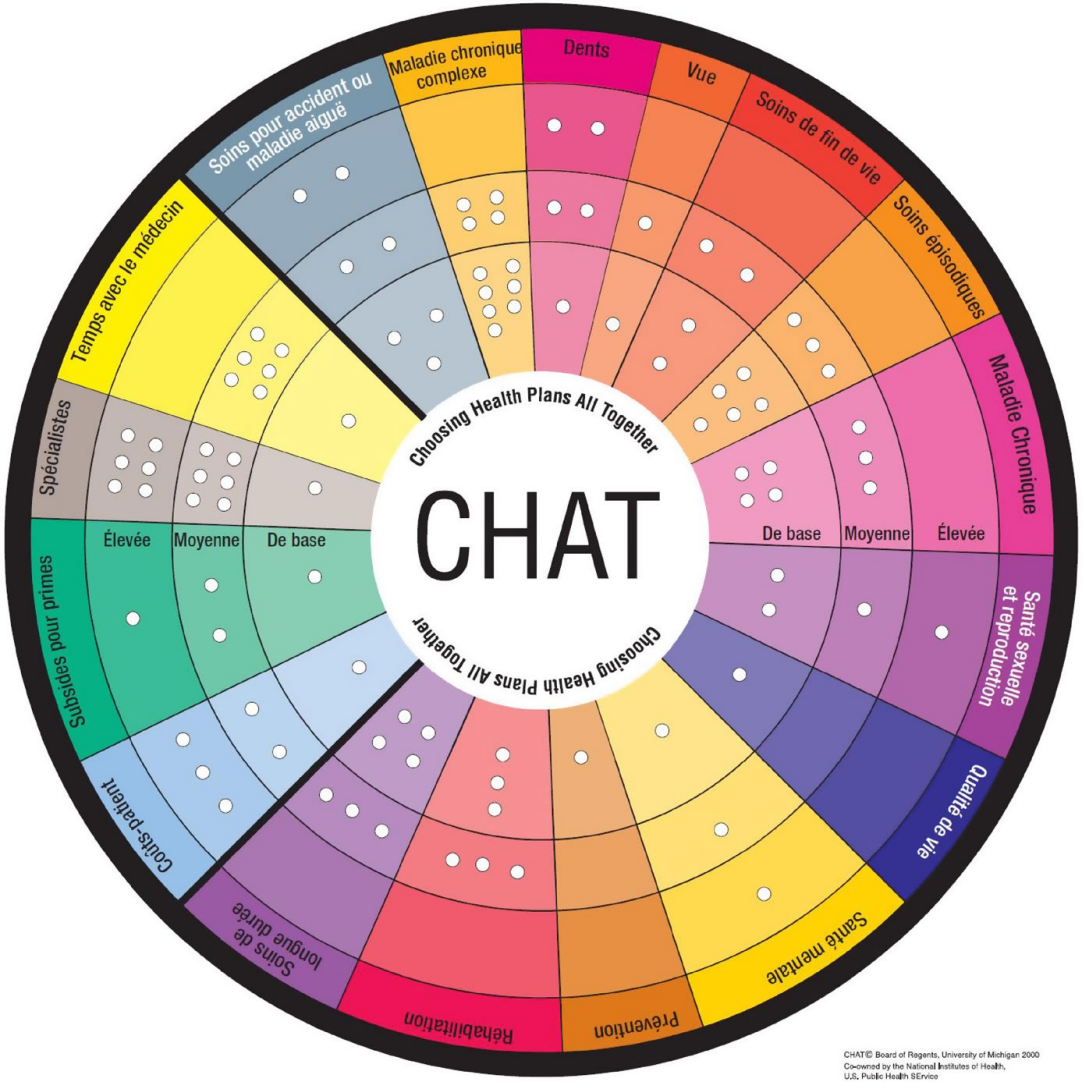
The Swiss‐CHAT board

The CHAT exercise is designed to allow participants to prioritize the type and level of health insurance benefits they prefer. It brings together small groups of people for approximately 3 hours and confronts people through a simulation exercise with the problem of prioritizing benefits to be covered by basic health insurance. Participants are expected to consider the choices they would make for their own sake and to deliberate about the best coverage for the entire population. To do this, participants use a pie‐shaped board on which the various benefit options (See Figure 1) are arrayed to make their choices. The board is shown in Figure 1 and the benefit options outlined in Box 1. We deliberately designed the board so that no single level would represent the Swiss status quo in every area. Participants are given 50 stickers representing units of currency to use in the selection of their benefit packages. Each sticker represents 1/50th of the average annual cost of health coverage for one person. Participants were guided in all rounds to first choose benefits at the basic level before selecting higher coverage levels. A CHAT manual written in simple language and describing the benefits for each coverage level, and the number of markers required to cover them, was also given to participants.

Box 1 **Domain descriptions**

1

**Table:** 

Optional categories
1. Severe injury or illness care: Care for sudden, bad injury or illness. Examples—sudden liver failure from food poisoning; massive injuries from an accident; a very premature and sick newborn.
2. Complicated Chronic Illness: Care of serious long illnesses like diabetes, heart failure, rheumatoid arthritis. These illnesses are complex and need lots of medical care to keep patients functioning as much as possible.
3. Dental: For care by dentists to prevent and treat dental problems. (Surgery of the jaw after injury, for example, is not here but under severe injury).
4. Vision: Testing and correcting for problems with eyesight that can be corrected with glasses or contact lens. Does not include other eye care. Laser treatment of the retina for diabetics would be covered by complex chronic illness.
5. End‐of‐life care: For patients with a terminal illness who are likely to die in a few months.
6. Episodic care: Treatment such as office visits, tests and drugs for short‐term problems, such as a sore knee, constipation, cough, heart burn or skin rash, but also short‐term urgent problems like appendicitis.
7. Chronic illness care: Routine checkups and care of chronic conditions that are new and not complicated.
8. Sexual and reproductive care: for care of birth control, pregnancy, sexual function and fertility.
9. Mental and behavioural care: For detecting and treating mental illness. May also cover behavioural health problems such as drug and alcohol abuse.
10. Quality of Life: For problems that are not badly disabling but affect quality of life, such as injuries affecting athletic performance. These problems affect a person's ability to act, look or feel well.
11. Prevention: To help prevent many diseases or illnesses. To identify medical problems as early as possible. There are no co‐pays for preventive services.
12. Rehabilitation: To restore or improve ability to do daily activities. This includes walking, speaking, bathing, eating and critical work functions. Often needed if a person has a stroke, a joint replaced or a limb removed.
13. Long‐term care: To pay for the care of a person who can no longer function independently that is provided at home or an institutional setting
Required categories
14. Out of pocket costs and premium: This is the money that individuals pay to use health‐care services. Co‐payments are not required for basic preventive services or routine screening tests
15. Premium subsidy: Subsidies given to lower income persons and families
16. Specialists: This is access to specialists and the range of choice of doctors and hospitals.
17. Time with the doctor: This is the frequency and length of medical visits.

Each group participated in four rounds of decision making. The first round required each person to choose a health plan that matched their own needs. In the second round, participants deliberated in groups of three or four to decide a collective health benefit plan for their canton. In the third round, all participants had to establish group consensus and choose an insurance plan together. In this third round, the moderator facilitated a group discussion by asking every person in turn to select a category of coverage (dental, vision, etc.), initially at the first level and subsequently at the second and third levels. The facilitator asked each participant to explain the reasons for his or her choice. Subsequently, group participants were asked whether or not they agreed with the selections made by individuals. This continued until all stickers were spent. In the fourth and final round, participants once again chose an insurance plan by themselves, using what they had learned in the previous cycles.

Before and after participation in the CHAT exercise, participants completed a survey to determine socio‐demographic characteristics and attitudes towards heath care. Their perception of the exercise was explored through two open‐ended questions in the post‐exercise survey. The questionnaires were established to understand the evolution of the participants' opinions on health benefits and basic health assurance. All materials were available in French, German and Italian.

### 2.5 Protection of human participants

Each participant was contacted and informed about the study and gave informed consent. Participants were paid 75 Swiss francs for their travel expenses and participation. This study was designated exempt from ethics review by the chair of the Geneva research ethics commission and the Office of Human Subjects Research Protection at the National Institutes of Health.

### 2.6 Data preparation and analysis

In this study, we report the results of qualitative analysis performed to understand the deliberative process and shed light on reasons put forward by participants to extend or deny coverage for different domains of health care in Switzerland. Group discussions were recorded, transcribed verbatim and translated into English (American Language Services, California, http://www.alsglobal.net) so that the research team could have a common language for analysing data.

Coding was based on content analysis, an approach considered appropriate “when existing theory or research literature on a phenomenon is limited” as was the case here.^13^ The first step involved examining the data, breaking them down and making comparisons and conceptualizations, which were then labelled with a set of codes. We then grouped codes into categories and distinguished groups of arguments used against or in favour of coverage for different domains of health care. For example, the following quotation: “Appendicitis is episodic care, a poisoning is episodic care, you can't wait for a month to do (sic) your appendicitis, waiting times can become long, and you need episodic care,” was initially coded as *Cannot wait for an appendicitis* simply to highlight the theme. Later, it became clear that a group of similar codes could be integrated into the category *Benefit for early intervention* and later in the more overarching theme of *Importance of medical care*. As categories emerged, we established a structured code list.

One principal coder (MS) coded all transcription and 20% of all the data were double coded by two separate coders (MD and SH) to ensure consistency and understanding of the use of the codes. Discrepancies between coders were settled through discussion between the three authors (MS, MD and SH). Questions regarding the intended meanings of words in the transcripts were settled by reference to the recordings by coders (MS and SH) who understood all three languages. Data saturation^14^, ^15^ was reached for the results reported here.

In the quotes presented here, additions are identified by []; where a part of the quote is left out, this is indicated by an ellipsis. For each quote, we indicate the focus group city, as well as the health domain and level of coverage discussed at that time.

## 3 RESULTS

### 3.1 Participants

Volunteers (N = 175) participated in 12 groups of 14‐16 individuals each. Participant characteristics are shown in Table 1. Despite being covered in a universal coverage health system, 20% of participants reported forgoing medical care for reasons of cost in the past twelve months.

**Table 1:** Participant characteristics

Age	
Median	44
Range	18‐88
Gender	
Male	46%
Female	54%
Language	
French	33%
German	50%
Italian	17%
Nationality	
Swiss	80%
Double	5%
European	10%
Other	5%
Marital status	
Married	39%
Single	25%
Partnered	15%
Divorced	20%
Widowed	1%
School level	
Primary	3%
Apprentice	38%
Secondary	9%
University	36%
Other	14%
Pay	
Mean	3000‐4999 CHF
Minimum	None
Maximum	>15'000 CHF
Health	
Excellent/VG	41%
Good	33%
Fair/Poor	27%

### 3.2 Reasons for and against coverage

Views put forward by participants in deciding which services to cover include strategic arguments and arguments for financial protection of individuals and families, attention to vulnerable persons (elderly, children or the mentally ill who might be stigmatized), for individual choice, cost‐benefit arguments including for the benefits of early intervention and primary care, avoiding redundant coverage, and arguments against excessive medicalization of life or the mechanization of medicine.

Analysis of the arguments, either in favour or against coverage, revealed the following overarching categories for inclusion of benefits (Table 2): strategy for coverage, financial arguments, identified groups, importance of medical care and responsibility argument.

**Table 2:** Arguments on priorities in health coverage

Code categories	Code list
Strategy for coverage (220)	Essential in a basic package (98)
	Acceptable trade‐off (27)
	Current level is insufficient (26)
	Additional insurance argument (21)
	Complete financial coverage (14)
	Alternative insurance argument (12)
	Another benefit is more important (9)
	Point argument (8)
	Lower level is justifiable (3)
	Redundancy (2)
Financial reasons (170)	Cost‐benefit argument (70)
	Importance of financial protection (32)
	Adverse effect of health costs (18)
	Protection against individual costs (17)
	Prevention for financial reason (13)
	Incomplete financial coverage (12)
	Argument about cost (8)
Protection of identified groups (91)	Importance of protection for identified groups (64)
	Attention to the elderly (13)
	Concern for family members (10)
	Difference between two groups (4)
Importance of medical care (91)	Benefit of early intervention (41)
	Prevention for health reason (23)
	Concern for treatment (10)
	Endorsement of triage by good doctors (9)
	General doctor argument (7)
	Appreciation of patient‐centred care (1)
The place of responsibility (61)	Responsibility for illness (extent or limitations) (20)
	Individual responsibility argument (15)
	Medical responsibility argument (14)
	Argument of individual choice (12)
Argument by example (52)	Personal experience argument (32)
	More severe disease deserves higher priority (15)
	The problem is not a health problem (5)
Collective argument (46)	Collective benefit argument (35)
	Importance of protection for everyone (11)
Considering disease factors (40)	Consequence of the disease (18)
	Real diseases should covered (13)
	Endorsement for comprehensive mental health (9)
Risk argument (36)	May cause another risk (24)
	Risk for life (12)
The quality of life factor (34)	Quality of life argument (27)
	Argument of well‐being of the work‐force (7)
The roles of insurance (22)	Consideration on insurance (14)
	Insurance coverage as a stop‐gap (6)
	Medicine can foster social inclusion (2)
Criticism of medicine and the health system (13)	Against merchandizing medicine (6)
	Criticism of consumerism (4)
	Criticism argument (2)
	Against medicalization (1)
Comparison argument (5)	Comparison between two countries (5)

**Table 3:** Participant perceptions of the CHAT exercise

Briefly, what (if anything)	
Did you find most valuable about doing CHAT?	Learned something (36)
	Heard the opinions of different people (32)
	Understood their own position better (13)
	Valued giving their own opinion, setting priorities and having influence (11)
	The discussion, argumentation and consensus (10)
	The discussion between generations (2)
	Going back to an individual plan at the end (2)
	People cared about the health system (2)
	People agreed on how expensive health care is (1)
	People were reasonable (1)
	The degree of consensus (1)
	Choices were difficult (1)
	Coverage is unequal (1)
	Everything is precious (1)
Surprised you most in today's session?	The diversity of opinions (28)
	It was constructive and interesting (10)
	The degree of consensus within the group (9)
	Choices were difficult (6)
	How little they and others understood before (6)
	Others were emotional or selfish (5)
	It was a game (4)
	The importance or unimportance of various domains to others (4)
	The examples presented by others and their importance (3)
	Essential cost‐saving mechanisms were not discussed during the exercise (3)
	Becoming aware of their role (2)
	Difference in costs between levels (2)
	That coverage was given to alcoholics and addicts (2)
	Becoming aware of implications within the health system (2)
	How much certain things cost (2)
	Others participated and changed their minds (2)
	Some remained opposed to vaccination (1)
	That they had learned something (1)
	That some could not afford care (1)
	The lack of data from health insurance (1)
	People want efficiency (1)

### 3.3 Coverage decisions can be strategic

“Strategy for coverage” was the most frequent argument given and the most frequently debated by participants. This included actions to achieve the goal that is to say the desired level of coverage. It includes the following codes: essential in a basic package, acceptable trade‐offs, current level is insufficient, additional insurance argument, complete financial coverage, alternative insurance argument, another benefit is more important, point argument, lower level is justifiable and redundancy.

Participants voiced a threshold view of health‐care coverage: if an element of coverage could be considered “basic,” then many participants considered that this implied that everyone should have access to it and thus that this was an argument to include it. Examples include:

So, I would really consider it important to include this [end of life] in the basic level. (Basel, essential in a basic package, end‐of‐life care, level 1)

It [mental health]is important on the basic level, as nowadays a lot of problems need psychological treatment. (Basel, mental health, level 1)

This is not purely cosmetic treatment, that's why I find it important to include it into the basic care. (Basel, vision, level 1)

Acceptable trade‐offs were often used as an argument in favour of the status quo, implying that all things considered the prevailing coverage was appropriate: additional coverage was then viewed as lacking sufficient importance to merit sacrificing something else.

This would be already covered by level 1. Apart from cases where you don't respond to treatments, the supporting treatment is covered. (Bern, accident or acute illness, level 2)

The most common codes articulated in opposition to coverage were as follows: another benefit is more important, acceptable trade‐off, alternative insurance is available. Here is an example of another benefit being more important:

So we'll retain the basic (level of coverage for vision). Because we're saving one point to place somewhere else. Placing 80 francs every five years would amount to nothing. We would save more by putting it somewhere else. (Biel, no vision, level 1)

Several participants mentioned the availability of other types of insurance (accident, disability, etc.), reflecting the value of coordination among various types of insurance, as a justification for their no coverage choices:

The reason why I don't want to include this in long‐term care is: what is included in the basic insurance? Because there is also the disability insurance! And a disabled child gets money from the disability insurance. Also, the disability insurance and the Swiss pension system pay for the aids for the elderly. It must also be considered that aids are paid for by the disability insurance and the Swiss pension system. This doesn't have to be included in the health insurance. This is a problem in Switzerland – one gets money from the disability insurance, one gets money from the Swiss pension system, and at the same time from the health insurance. I am in favor of long‐term care, but we must be careful not to let it become an over‐insurance, because, as I said, both the disability insurance and the Swiss pension system provide funds, and aids can be purchased (Chur, no episodic care, level 1)

### 3.4 Financial reasons

“Financial arguments” were the second most frequently used category of arguments and the most discussed by the participants. It included cost‐benefit, the importance of financial protection, adverse effects of health costs, protection against individual costs, prevention for financial reason, incomplete financial coverage and other arguments about cost.

For several participants, the cost‐benefit argument (weighing costs with the benefits of an intervention) was an important consideration in favour of coverage. Participants weighed the impact of investing stickers in different domains and compared them.

We have to consider which points can be set cheaply and which points are expensive, because if you see… For example, if we take (dental care), I would also like to go to the second level, but if you're realistic, then dental hygiene and a couple of x‐rays per year can't be compared to the costs for the three points under “Long‐term care”, and I think this would be worth discussion, which points are cheap, so to speak, and for which points you have to pay a lot. (St‐Gall, long term care, level 1)

Participants sometimes viewed interventions in a manner similar to a financial investment. Here is an example of such an argument given in favour of insuring interventions that improve quality of life.

You have to consider the costs that are created if somebody's quality of life is affected! Those are the instances where, in the most extreme cases, persons can't contribute to the insurance any more. And by treasuring the quality of life a little bit, we can prevent this from happening. This is my opinion. (Chur, quality of life, level 1)

In contrast, some participants do not go to doctors, especially dentists, for economic reasons.

Because I see that in Switzerland, a lot of people don't go to the dentist for years, because the check‐up alone costs 200 francs, and they have to pay for it themselves, so the maximum amount, 1,000 francs, wouldn't be enough for anything. If people didn't go to a dentist for 10 years, and then they finally do go, then they have so many holes and so much calculus that those 1,000 francs aren't enough. (St‐Gallen, dental, level 2)

### 3.5 Importance of identified groups

Participants argued on behalf of several groups of individuals as vulnerable and worthy of priority including families, seniors, children, diabetics, pregnant woman, mentally ill persons, teenager, young couples and workers. Codes in this category included importance of protection for identified groups, attention to the elderly, concern for family members and difference between two groups. Here is an example for families:

Because it says that it would include even financial support for family members, and that is in my opinion a very important point. Because you easily forget how much care and time a mentally ill person requires. It is more than a full‐time job. It is indeed a 24/7‐job. And you can't really work another job. So I think it is very important to have this kind of support included in there. I would choose level 3. (Zurich, mental illness, level 3)

For participants, there was a consensus regarding issues affecting families. They considered the family a unit that must be particularly protected. One respondent gave this example:

Alcoholism and drug use is also important, but I think for that we should be able to have it treated. It should be covered because it can destroy families. It can bring about even higher costs. It can make the rest of the family sick. An alcoholic at home can also make the rest of the family sick, but other illnesses… psychological illnesses. When you have children, it's terrible. So I think it's important. (Biel, mental health, level 3)

During discussion, whenever anyone spoke of her own story in the first person, participants took such statements very seriously as a justification for solidarity. Here is such an example:

Well I'm a 20 years old girl and I'm doing my internship and I'm pregnant, and I'm honestly more than happy that at least the basic is covered, because being an intern, I would never have the money to pay every month every time I have to go to the gynecologist, the ultrasound studies, that are also useful to look at, and I'm happy with the basic package. That has to be in the basic at least. (Lugano, sexual health, level 2)

Thank you. Anyone else? Anyone else who has to say something or else we vote.

One last thing, besides the 9 echographies that need to be done, I don't know, I don't have kids, I'm not pregnant, but if I would be pregnant, I wouldn't do 1 per month, I would do 4 per month for the way I am, but that would be an excessive cost. But, it's based on the mother's health when she's pregnant; therefore if there is something that's not ok with the baby, it goes back to the mother. With this said, I am voting yes for the basic, but at this point even a step higher, medium, because if there is a minimal problem, the baby and the mother will both risk their lives. (Lugano, sexual health, level 2)

### 3.6 Importance of medical care

“Importance of medical care” is a category that focuses on the importance of taking care of health problems. It includes benefit for early intervention, prevention, endorsement of triage by capable doctors, argument about general doctor and appreciation of patient‐centred care. Here is an example of a benefit for early intervention:

One can also see that… Well, those are special treatments, specialized clinics, and if a depression is treated well at an early stage, then there is a better chance that it won't come back (…) (Basel, mental health, level 2)

Some participants strongly endorsed triage by capable doctors, being seen initially by trustworthy professionals, as a very important component of high‐quality medical care. It is important that the first doctor to see a patient's problem be effective.

But doctors are not just doctors…they are specialists, surgeons, etc. They are not just general doctors. Let's say you are at the hospital, and you have received emergency care, the doctor will not look at his watch, he will do what is necessary.(Sion, endorsement of triage by good doctors, level 2)

The importance of having a family doctor can be an argument for forgoing coverage of direct access to specialists. It is important for episodic care but also to direct people when they need to go to a specialist and for long‐term monitoring of people.

Well let's discuss one thing. Because when you assign these six points to specialists, it means that a person can go see a dozen until they get the right answer. I am for the family doctor, which is no longer being done because there is not enough money from insurers. In the past, the family doctor would say “I will send you there”. Now, no one has a family doctor. There are many young people who do not have doctors. I think that logically and for full health, I think that a family doctor is worth more than what insurers are offering. That is an opinion. (Lausanne, No specialists, level 2)

### 3.7 Struggling with the place of responsibility

The “Responsibility argument” category included arguments regarding the causal role of individual behaviour and medical care. The question of individual responsibility for health problems recurred frequently as a source of controversy in discussions. Some people thought that we are not responsible for their health problems and the need to ensure coverage:

It's not your fault if you have a chronic illness. (Lausanne, chronic diseases, level 1)

Arguments related to responsibility, particularly individual responsibility, were very often articulated as a justification for not selecting coverage.

Everybody goes to the dentists with their cavities to have them repaired, and the problem is: what was the reason their teeth got bad? Of course, in a lot of cases it has genetic reasons, but a lot of people ruin their teeth by consuming too much sugar, or carbohydrates, or all those poisons. I don't approve of this being covered by the insurance, as well. (Basel, individual responsibility argument, no dental, level 1)

While others argued not to pay for problems that would be associated with risky behaviours of individuals:

Yes, well in that case, they could pay attention to self‐medication. People could stop smoking; people could stop eating poorly… (Geneva, subsidies for premiums, level 2)

### 3.8 Participant perceptions of the CHAT exercise

In their short open‐ended answers for the post‐exercise questionnaire, participants reported that the game made it easy to understand information (94%) and to make decisions (70%) and that they were satisfied with the group choice (91%) (Table 3). They also reported what they found most valuable and most surprising about the exercise.

The aspects reported as most valuable by participants were that they had learned something, heard the opinions of different people and understood their own position better. They valued giving their own opinion, setting priorities in the exercise and having influence. Participants reported being most surprised by the diversity of opinions, by how constructive and interesting the game was, and by the degree of consensus reached. General remarks included requests for the results, and one comment that this was better than voting.

## 4 DISCUSSION

In a context with universal health coverage under an individual insurance mandate with a strong habit of participation in public decisions, participants' reasons for prioritizing certain forms of coverage included an effectively managed health system with basic coverage for all and special protections for vulnerable groups. Participants were particularly attentive to the costs of health care and means of reducing these costs. They also focused on subsidiarity and systemic thinking. They developed strategic arguments with a view for the best financial management. They avoided duplication, treated some forms of coverage as an investment intended to prevent further costs and provided indirect assistance for families to enable them to remain the first support of the sick. We also found arguments grounded in solidarity: the importance of protection for everyone, specifically protection for vulnerable groups, the importance of benefitting families and the importance of personal stories to shaping decisions. Individual responsibility was considered a component of solidarity: part of individuals' share in contributing to a health‐care system responsive to all. In this study conducted among a sample of Swiss residents, participants were also able to become actively involved in difficult trade‐offs regarding health coverage and set priorities in a health system with limited resources.

Although there are several articles that exploring the possibility of a decrease in solidarity in Switzerland and elsewhere following social health insurance reform,^16^, ^17^, ^18^ our results show persistence of a strong and practical notion of solidarity as reflected by the priority placed on providing basic protection for everyone. When a level of coverage was described as basic, however, participants initially considered including it as the default. They did exclude some services from coverage altogether, such as quality of life and sometimes prevention, but this required deliberation. Participants also prioritized protection of groups they considered most vulnerable, particularly families, children, elderly persons, diabetics, pregnant women and mentally ill individuals. Arguments for protecting families included expectations that they would in turn protect sick individuals, for example in case of chronic of mental illness. The argument based on personal experience of the type “in my family, we lived as…” brought more consensus than abstract examples. Here, we see the forms of solidarity linked to family needs and to contextualized personal experience. This view of solidarity is not one where help through families and help through insurance were in opposition. The relation between them is one of subsidiarity or complementarity. Help ought to come from families first but when families exhaust their ability to help, insurance should step in. Insurance should also help families to remain able to help their members.

The effectiveness of the management system was considered essential. Participants recognized a high probability of increasing costs, considered some coverage to be a form of investment, to prevent further expenses later on and took great care to avoid duplication. The effectiveness of care and support was also very important to our participants. Triage by a competent doctor was considered crucial to judge the seriousness of an illness episode and to determine whether referral to a specialist is necessary. Most participants considered the family physician to be most important for the coordination and efficiency of care. Although some did value direct access to specialists, most considered that family physicians had all the necessary abilities to guide patients, ensure their overall health and follow them over the long‐term.

There is a long‐standing debate about the nature and implications of personal responsibility for health,^19^, ^20^ and this topic proved a challenge to our participants as well. Our findings suggest that there was a tension between the lack of fault for having a disease, and the need for accountability for health behaviour that influences solidarity and rising health costs. Making an effort to pursue healthy behaviour was perceived as a demonstration of solidarity on the part of an individual—it helps contain costs and facilitates more affordable treatment for everyone.

Our study has several limitations. Fundamentally, this was a hypothetical exercise. Participants, however, remarked that results could be brought to bear on real decisions in the Swiss context and this could have decreased the distance between choices made here and choices they would have made in practice. The participants in this research are not a representative sample in the statistical sense. The frequency of their responses cannot be interpreted as representing the prevalence of these views within the Swiss population. Instead, our sample represents the diversity of profiles of people living in Switzerland and thus the arguments we found should approximate the range of arguments one might find in the Swiss population. Use of an agency that usually recruits participants for market research may have meant that our participants were over selected for their interest in market research. However, recruitment intentionally included diverse socio‐economic profiles and our demographic data suggest that this was successful. While we were not able to select on the basis of health status or experiences of illness, our sample did include people with a range of health states. As the views of both the sick and healthy are relevant to decisions regarding health‐care coverage, homogenous groups of either sick or healthy participants would have weakened our groups' ability to recognize some important trade‐offs. Despite lack of stratification for health status, we did obtain both sick and healthy participants in our groups. We paid participants and this could have led to an overrepresentation of participants in need of the money. For this reason, recruitment was targeted to obtain a diversity of economic levels and here too our demographic data indicate that this was successful. We translated our material into English to have a common language for the coding phase, and this may have modified nuances and influenced results. This is unlikely to have affected the main structural components presented here, as they occurred repeatedly with different phraseology in all three study languages. Moreover, as the research team included members fluent in all the study languages, checks were conducted against the original version when clarification or confirmation was required. As is usual with qualitative methodology, any generalizations to different countries and health systems must be cautious. We used a highly structured focus group methodology and this may have influenced some of the responses. This was necessary, on the other hand, to explore specific trade‐offs as this required that participants focus on these choices rather than providing us with only more general views of how priorities ought to be set in the health system.

Our findings regarding the issues raised by participants—as well as their own perception of what was valuable during discussion—confirm that this exercise fosters deliberation and enables ordinary citizens to balance individual priorities and collective responsibilities.^11^, ^21^ Rather than limiting public input to either outlining general principles or commenting on specific interventions,^3^ this process enabled the emergence of general principles for priority‐setting based on a trade‐off exercise by citizens. This exercise revealed several levels of reflection among the participants. They made priority‐setting decisions, but also addressed more general moral issues associated with solidarity and responsibility. Our participants did not consider personal responsibility to be a straightforward matter of personal fault, justifying lesser coverage. Rather, they considered individual responsibility to be a component of solidarity: healthy behaviour was part of individuals' share in contributing to a health‐care system responsive to all. From this angle, responsibility for health does not justify punishment if a non‐responsible action is performed, but is rather something to internalize to maintain solidarity for all. Cost‐effectiveness was integrated with solidarity as well. Efficient management of the health system was considered one of the means to ensure basic coverage for all and special protections for vulnerable groups.

The Swiss‐CHAT exercise created conditions for reflection and deliberation, enabling participants to think through trade‐offs more complex than the “yes/no” questions presented during public referenda. Findings supporting a similar conclusion were also reported following citizen deliberation in the United States and India.^21^ Although the Swiss population is particularly accustomed to participatory democracy, this exercise has been used elsewhere with similar effectiveness in promoting deliberation and group engagement. Our results suggest that the capabilities of the general public to take part in setting priorities on complex issues should not be underestimated. These issues are likely to become yet more pressing with developments in personalized medicine and increasing costs of biologics and cancer drugs.^22^ At a time when European health‐care systems are poised to face further increases in health‐care costs in a context of important economic challenges, public engagement placing trade‐offs on the table may be more feasible than one might think.

## NOTE

1 Choosing Healthplans All Together: http://www.chat-health.org
